# Oral administration of *Pantoea agglomerans*-derived lipopolysaccharide prevents metabolic dysfunction and Alzheimer’s disease-related memory loss in senescence-accelerated prone 8 (SAMP8) mice fed a high-fat diet

**DOI:** 10.1371/journal.pone.0198493

**Published:** 2018-06-01

**Authors:** Yutaro Kobayashi, Hiroyuki Inagawa, Chie Kohchi, Kimiko Kazumura, Hiroshi Tsuchiya, Toshiyuki Miwa, Katsuichiro Okazaki, Gen-Ichiro Soma

**Affiliations:** 1 Departments of Integrated and Holistic Immunology, Faculty of Medicine, Kagawa University, Kagawa, Japan; 2 Control of Innate Immunity, Technology Research Association, Kagawa, Japan; 3 Research Institute for Healthy Living, Niigata University of Pharmacy and Applied Life Sciences, Niigata, Japan; 4 Macrophi Inc., Kagawa, Japan; 5 Central Research Laboratory, Hamamatsu Photonics K.K., Shizuoka, Japan; 6 Department of Applied Biological Science, Faculty of Agriculture, Kagawa University, Kagawa, Japan; The University of Tokyo, JAPAN

## Abstract

The pathogenesis of Alzheimer’s disease (AD) remains unclear, but an imbalance between the production and clearance of amyloid-β (Aβ) peptides is known to play a critical role in AD progression. A promising preventative approach is to enhance the normal Aβ clearance activity of brain phagocytes such as microglia. In mice, the intraperitoneal injection of Toll-like receptor 4 agonist was shown to enhance Aβ clearance and exhibit a preventative effect on AD-related pathology. Our previous clinical study demonstrated that orally administered *Pantoea agglomerans*-derived lipopolysaccharide (LPSp) exhibited an LDL (low-density lipoprotein)-lowering effect in human volunteers with hyperlipidemia, a known risk factor for AD. *In vitro* studies have shown that LPSp treatment increases Aβ phagocytosis by microglial cells; however it is still unclear whether orally administered LPSp exhibits a preventive effect on AD progression. We show here that in senescence-accelerated prone 8 (SAMP8) mice fed a high-fat diet, oral administration of LPSp at 0.3 or 1 mg/kg body weight·day for 18 weeks significantly improved glucose metabolism and lipid profiles. The LPSp treatment also reduced pro-inflammatory cytokine expression and oxidative-burst activity in the peripheral blood. Moreover, LPSp significantly reduced brain Aβ burden and memory impairment as seen in the water maze test, although we could not confirm a significant enhancement of Aβ phagocytosis in microglia isolated from the brains after treatment. Taken together, our results show that LPSp holds promise as a preventative therapy for AD or AD-related diseases induced by impairment of metabolic functions.

## Introduction

Alzheimer’s disease (AD) is an age-dependent neurodegenerative pathology characterized by irreversible and progressive dysfunction of cognition and behavior. The primary pathological features of AD occur in brain parenchyma: progressive deposition of amyloid β (Aβ) peptides derived from Aβ protein precursor (APP) and aggregation of hyperphosphorylated tau protein [1]. The pathogenesis of AD remains unclear, but one widely accepted hypothesis, known as the amyloid cascade hypothesis, postulates that AD neurodegeneration is caused by an imbalance between production and clearance of Aβ peptides in the central nervous system [2]. β- and γ-Secretases have been identified as the proteases responsible for generating Aβ species by cleavage of APP, and are thus considered a prime therapeutic target in AD. Many secretase inhibitors have been designed and synthesized, but no efficient clinical drug has emerged until now due to the presence of side effects in humans [3].

An alternative and promising approach is to enhance Aβ clearance by brain-resident phagocytes such as microglia. The microglial phagocytic response plays a central role in removing dead cells, cellular debris, and Aβ peptides, and contributes to brain homeostasis. Toll-like receptor 4 (TLR4), as well as TLR2 and CD14, are essential to enhancing a phagocytic response by microglial cells directed at Aβ [4]. Importantly, recent *in vivo* studies using a transgenic mouse model of AD have demonstrated a preventive effect on AD development resulting from mild TLR4 stimulation with insufficient to induce obvious inflammation. The intraperitoneal (i.p.) injection of lipopolysaccharides (LPS) at a low dose (0.15 mg/kg body weight [BW] ·week, 12 weeks) was found to reduce the cerebral content of phosphorylated tau protein, implying a TLR4-mediated degradation of phosphorylated tau without alteration of inflammatory-cytokine gene expression [5]. Monophosphoryl lipid A (MPL) derived from *Salmonella minnesota* R595 LPS enhanced a phagocytic response against Aβ_1–42_ peptides in a mouse microglial cell line and i.p. administration of MPL prevented brain Aβ burden and enhanced cognitive function [6].

Our previous studies have demonstrated beneficial effects of orally administered LPSp (LPS derived from *Pantoea agglomerans*) on immune-related diseases. LPSp was originally found in a water extract of wheat flour, which produced moderate macrophage activation in mice [7]. Oral administration of LPSp (1 mg/kg BW·day, 1 week) to mice was found to enhance a phagocytic activity dependent on the TLR4 signaling pathway in the resident peritoneal macrophage with no augmentation of inflammatory cytokine production [8]. High dose of LPSp (300 mg/kg BW·day, 4 weeks) caused no significant hepatotoxicity or nephrotoxicity when orally treated to rats [9]. In addition, other *in vivo* studies showed that oral administration of LPSp or LPS derived from wheat flour exerts a preventive effect on atopic dermatitis [10], diabetes mellitus [11] and hyperlipidemia [12]. Other researchers demonstrated that sublingual administration of LPSp to mice resulted in TLR4-dependent activation of local innate immune cells in the cervical lymph nodes, which enhanced systemic resistance to pathogen infection [13]. Our previous clinical study reported that oral administration of an LPSp-containing tea resulted in a significant reduction in levels of fasting plasma glucose and low-density lipoprotein (LDL) cholesterol in hyperlipidemic volunteers, with no adverse effects [14]. Many epidemiological data [15] support the association of cardiovascular risk factors (diabetes mellitus, hyperlipidemia, and hypertension) with AD pathogenesis such as amyloidosis and cognitive decline, suggesting an impairment of glucose/lipid metabolism contributes to AD by accelerating an oxidative-stress and inflammatory response, leading to Aβ deposition and neural dysfunction. Our recent *in vivo* study demonstrated that oral administration of LPSp prevented the development of atherosclerosis *via* reducing hyperlipidemia, pro-inflammatory mediators and oxidative responses in high-fat diet (HFD)-fed mice [16]. Other studies also reported that oral treatment with a heat shock protein (HSP; known as a TLR4 agonist) prevents the development of diabetes mellitus and hyperlipidemia by improving glucose/lipid metabolism and reducing inflammatory responses in mice [17, 18], however few studies have shown a preventive effect on AD progression by orally administered TLR4 agonists. Our *in vitro* studies have demonstrated that LPSp significantly increases phagocytic activity directed at Aβ_1–42_ peptides in a mouse microglial cell line and primary microglia isolated from mouse brain [19, 20]. This evidence prompted us to consider whether LPSp may exert an *in vivo* preventative effect on AD development.

Therefore, we here investigated whether oral administration of LPSp is effective in ameliorating the development of AD-related symptoms in a mouse model of AD, the senescence-accelerated prone 8 (SAMP8) mice with fed a HFD. This model has been demonstrated to exhibit AD-like pathology and cognitive deficits in parallel with the development of diabetes [21]. Orally administered LPSp for 18 weeks significantly reduced fasting blood glucose level, plasma LDL, plasma oxidized LDL (ox-LDL) level, pro-inflammatory mediators, and the oxidative burst activities in the peripheral blood sample. In addition, LPSp significantly reduced brain Aβ accumulation and memory impairment as seen in the water maze test, although we could not confirm an enhancement of Aβ phagocytosis in microglia isolated from the brains after treatment. Our findings firstly demonstrated that LPSp can effectively ameliorate hyperlipidemia and inflammatory/oxidative responses to prevent AD progression and AD-related memory loss when orally administered to HFD-fed SAMP8 mice.

## Materials and methods

### Animals

**Fig 1:**
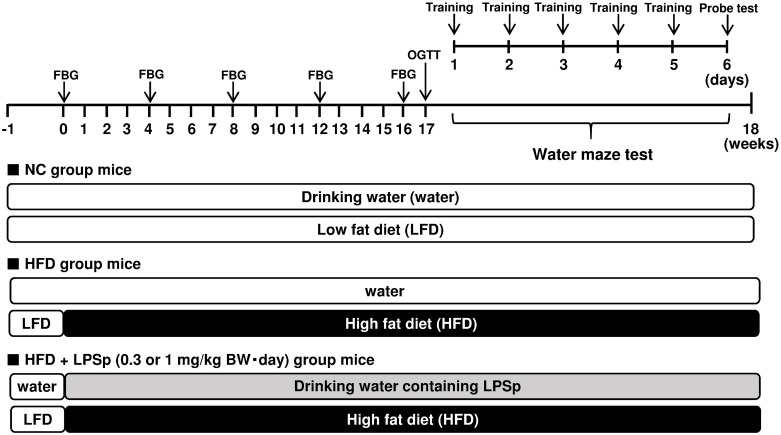
Experimental design and schedule. FBG, fasting blood glucose test; OGTT, oral glucose tolerance test; LPSp, lipopolysaccharide from *Pantoea agglomerans*; BW, body weight.

Male SAMP8 mice, aged 12–14 weeks, were obtained from Japan SLC, Inc. (Shizuoka, Japan) and housed under conditions of controlled temperature and humidity with a 12-h light/dark cycle and unrestricted access to food and water. A low-fat diet (LFD; 16.1 kJ/g, 10% of energy as fat, D12450B) and a high-fat diet (HFD; 21.9 kJ/g, 60% of energy as fat, D12492) were purchased from Research Diets, Inc. (New Brunswick, NJ, USA). All mice were acclimated for 1 week to an LFD and tap water. The experimental schedule is summarized in Fig 1. Mice were fed HFD to induce the development of experimental AD. Purified LPS derived from *P*. *agglomerans* (LPSp; Macrophi Inc., Kagawa, Japan) was dissolved in drinking water and applied at 0.3 or 1 mg/kg BW·day for 18 weeks (*n* = 7 both groups). The drinking water was changed weekly, and the concentration of LPSp was adjusted according to the average BW and amount of water consumption. The dose of LPSp was determined to be the sufficient dose required to achieve preventive effects as estimated from previous studies (0.1–1 mg/kg BW/day) [8, 10, 16]. BW, food intake, and water intake were recorded weekly. The NC group received LFD and water only (*n* = 4), and the positive control (HFD) group received HFD and water only (*n* = 6) during the experiment. At the end of week 18, the mice were anesthetized under sevoflurane vapor after overnight food deprivation. Blood samples were collected via heart puncture with a syringe rinsed with heparin. The whole blood samples were used for further analysis within 6 h after collection. Plasma was collected after centrifugation (1,200 × *g* for 20 min at 4°C) and stored at −80°C. Liver and epididymal white adipose tissues were removed and weighed. Whole brain tissue was excised, and the brain hemispheres were either frozen at −80°C for Aβs ELISA analysis or kept in phosphate buffered saline (PBS; Sigma Aldrich Co. LLC, St. Louis, MO, USA) for measurement of phagocytic activity against Aβ_1–42_. The colon tissue was removed and stored at −80°C until use. The experiment protocols were approved by the Animal Care and Use Committee of Kagawa University (approval no. 15154). This experiment was carried out according to the guidelines for animal experiments of the Faculty of Medicine, Kagawa University.

### Measurement of fasting blood glucose and oral glucose tolerance test

The fasting blood glucose (FBG) level was measured every 4th week (week 0, 4, 8, 12, and 16) using an Accu-Chek Aviva blood glucose meter with Accu-Chek Aviva test strips (Roche Diagnostics K.K., Quebec, Canada) with tail-vein blood collected from overnight-fasted mice. At week 17, the mice were fasted overnight and administered an oral glucose tolerance test (OGTT) by gavage with 2 g of _D_-glucose/kg BW. Blood samples were collected from the tail vein, and blood glucose levels were determined at 0, 15, 30, 60, and 120 min after glucose loading. The area under the curve (AUC) was calculated using the trapezoid rule.

### Biochemical measurements

Plasma insulin levels were measured using a mouse-insulin ELISA kit (AKRIN-011S; Shibayagi Co., Ltd., Gunma, Japan) in accordance with the manufacturer’s instructions. Hemoglobin A1c (HbA1c) was determined using an HbA1c measurement kit (Sekisui Medical Co., Ltd., Tokyo, Japan). Plasma triglycerides, LDL, high-density lipoprotein (HDL), total cholesterol, non-esterified cholesterol levels, AST (aspartate transaminase), and ALT (alanine transaminase) were measured using commercial kits (Wako Pure Chemical Industries). Plasma ox-LDL was determined with a mouse Ox-LDL ELISA kit (Kamiya Biomedical Company, LLC, Tukwila, WA, USA). Lipid extraction from the liver tissues was performed with the butanol-methanol method as described previously [22] and the lipid extracts were stored at −80°C until use. Hepatic triglyceride levels were measured with a triglyceride measurement kit (Wako Pure Chemical Industries) and are reported as mg of triglyceride per g of liver weight. The plasma cytokines were determined using commercial mouse ELISA kits (Affymetrix Inc., Santa Clara, CA, USA), catalog numbers as follows: tumor necrosis factor (TNF)-α (#88–7324) and interleukin (IL)-6 (#88–7064). The frozen colon tissue was homogenized in ice-cold PBS containing 1% protease inhibitor cocktail (GE Healthcare UK Ltd., Buckinghamshire, England) for 3 min using a homogenizer. The supernatant was collected following centrifugation at 10,000 × *g* for 10 min at 4°C. The cytokines in the colon tissue were determined using a following mouse ELISA kit according to the manufacturer’s instructions: TNF-α (#88–7324), IL-6 (#88–7064) and IL-10 (#88–7105, Affymetrix Inc.). The concentration of colonic cytokines is reported as picograms of cytokine relative to protein content (mg of protein), the latter determined by a bicinchoninic acid (BCA) assay (Thermo Fisher Scientific, Waltham, MA, USA). The total abundance of LPS in plasma was analyzed by a kinetic turbidimetric assay using the Limulus Amebocyte Lysate (LAL) assay kit and a Toxinometer ET-6000 computer-operated kinetic incubating tube reader (Wako Pure Chemical Industries) according to the manufacturer’s instructions, with the following modifications: the plasma was diluted 1:10 in pyrogen-free water (Otsuka Pharmaceutical Factory, Inc., Tokushima, Japan) and preheated to 70°C for 10 min prior to analysis.

### Simultaneous measurement of superoxide anion (O_2_^·-^) and myeloperoxidase (MPO) activity by real-time monitoring of chemiluminescence and fluorescence

O_2_^**·**-^ production and MPO activity in the peripheral blood samples were simultaneously determined after stimulation with phorbol 12-myristate 13-acetate (PMA; Wako Pure Chemical Industries) using a real-time chemiluminescence and fluorescence monitoring system (CFL-P2200; Hamamatsu Photonics K.K., Shizuoka, Japan), as previously described [16]. Briefly, an aliquot of each whole blood sample (30 μl) was incubated with 500 μl of an ammonium chloride-based red-blood-cell lysis buffer with no fixing reagents (Tonbo Biosciences, Inc., San Diego, CA, USA) for 2 min at room temperature (RT), and the cells were then pelleted by centrifugation (200 × *g* for 5 min at RT). The white-blood-cell pellet was suspended in Ringer-Hepes buffer (154 mM NaCl, 5.6 mM KCl, and 10 mM Hepes, pH 7.4) and pre-incubated with 1 mM CaCl_2_, 0.5 μM MCLA (6-(4-methoxyphenyl)-2-methyl-3,7-dihydroimidazo[1,2-a]pyrazin-3-one hydrochloride; Tokyo Chemical Industry Co., Ltd., Tokyo, Japan), and 2 μM aminophenyl fluorescein (APF; Goryo Chemical, Inc., Hokkaido, Japan) on a glass slide for 2 min at 37°C. Here, MCLA was used as an O_2_^**·**-^-sensitive chemiluminescence probe and APF was used as a hypochlorous acid-sensitive fluorescence probe. To confirm the specificity of these probes for O_2_^**·**-^ and hypochlorous acid in the PMA-stimulation experiment, 9 μg/ml of superoxide dismutase (SOD; Sigma Aldrich; a potent scavenger for O_2_^**·**-^) and 100 μM of 4-aminobenzoic acid hydrazide (4-ABAH; Sigma Aldrich; a potent inhibitor of MPO-mediated hypochlorous acid production) were added to the sample 3 min before the addition of PMA. The chemiluminescence intensity was calculated as relative chemiluminescence units (RCU) and the fluorescence intensity as relative fluorescence units (RFU) at a sampling rate (0.5 s). The sample was continuously stirred at 37°C during the measurements. After 60 s, PMA (1 μM) was injected into the sample by auto injector. The PMA-induced responses were calculated as the peak intensity minus the basal value.

### Spatial learning and memory assessment

To assess spatial learning and memory, we administered the Morris water maze (MWM) test at week 17 as described previously [23]. The experimental apparatus consisted of a circular tank 100 cm in diameter and 40 cm in height filled with water to 30 cm, maintained at 23 ± 1°C, and rendered opaque with non-toxic black ink. The area of the pool was conceptually divided into four equal quadrants, and a different black shape cardboard was placed on the wall of each quadrant. A removable circular platform 10 cm in diameter was submerged 1 cm below the water surface and placed approximately in the midpoint of one quadrant, defined as the target quadrant. A video camera was hung above the center of the pool to record the swimming paths of the mice. In the present study, each mouse consecutively received a pre-training session (1 day), training sessions (5 days), and a probe session (1 day) as follows. One day before the first training session, each mouse received a pre-training session to make it aware of the escape platform. The mouse was put on the platform for 20 s, given a 30-s free swim, and then assisted in swimming back to the platform. The next day, the first training session was conducted to assess spatial learning ability. Training sessions continued for five consecutive days. On each trial, the mouse was released into the water at a randomly assigned starting position out of three possibilities, facing the pool’s wall. Mice were given 60 s to find the platform and were allowed to stay on it for 20 s. The spatial learning ability of each mouse was evaluated by the amount of elapsed time, defined as escape latency. If the mouse failed to find the platform within 60 s, it was gently guided to the platform and kept there for 20 s, and the escape latency was recorded as 60 s. Each mouse underwent four trials per day, and the average value of the escape latency was calculated. Next day after the last training session, the probe test was performed to assess the spatial memory ability of the mice as follows. The platform was removed from the pool, and each mouse was placed in the pool at a starting position located opposite the target quadrant and allowed to swim freely for 60 s. The time spent swimming in the target quadrant was recorded. The swimming trajectory was visualized using AnimalTracker software [24].

### Determination of Aβ_1–40_ and Aβ_1–42_ in the mouse brain

The levels of Aβ_1–40_ and Aβ_1–42_ in both the soluble and insoluble fractions were determined using an ELISA kit (#294–64701, Aβ_1–40_ and #292–64501, Aβ_1–42_; Wako Pure Chemical Industries) in accordance with the manufacturer’s instructions. The snap-frozen brain were homogenized in ice-cold RIPA (radioimmunoprecipitation assay) buffer (Nacalai Tesque, Inc., Kyoto, Japan) containing 1% protease inhibitor cocktail and 1 × PhosSTOP (Roche Diagnostics GMbH, Basel, Switzerland), followed by centrifugation at 100,000 × *g* for 1 h at 4°C. The supernatant was collected for further analysis of soluble Aβ peptides. Then the pellet was extracted in ice-cold 70% (v/v) formic acid (Wako Pure Chemical Industries) followed by centrifugation at 100,000 × *g* for 1 h at 4°C. The supernatant was neutralized with a 20-fold dilution in 1M Tris buffer and analyzed for insoluble Aβ peptides. The protein concentration of individual fractions was measured by BCA assay (Thermo Fisher Scientific).

### Flow-cytometric analysis of phagocytic activity against Aβ1–42 by isolated brain microglial cells

Microglial cells were isolated from the brain by enzymatic digestion, as previously described [20]. Briefly, the brain homogenate was incubated in serum-free Dulbecco’s modified Eagle’s medium (DMEM; Sigma Aldrich) containing 1.2 units/ml dispase II, 1 mg/ml papain (Sigma Aldrich), and 10 units/ml DNase I (Takara Bio Inc., Shiga, Japan), for 30 min at 37°C. The enzymatic digestion was stopped by the addition of DMEM containing 10% fetal bovine serum (FBS; Sigma Aldrich) followed by centrifugation for 5 min at 300 × *g* at RT. The cells were incubated with magnetic myelin removal beads and anti-CD11b antibodies conjugated to magnetic beads (Miltenyi Biotec GmbH, Bergisch Gladbach, Germany). The CD11b^+^ cells were isolated using an autoMACS^®^ pro separator (Miltenyi Biotec). The isolated CD11b^+^ cells were plated for 24 h in poly-_D_-lysine–coated 96-well plates in DMEM medium containing 10% FBS at 37°C. The medium was then replaced with serum-free DMEM containing HiLyte^™^ Fluor 488-Aβ_1–42_ (HF488-Aβ_1–42_; Anaspec Inc., Fremont, CA, USA) at a concentration of 0.5 μg/well and incubated for 3 h at 37°C. To exclude the non-specific fluorescence of non-internalized HF488-Aβ_1–42_, a control was included in which cytochalasin D (CyD; Wako Pure Chemical Industries), a potent inhibitor of phagocytosis, was added at 10 μM to the cells 30 min before adding the HF488-Aβ_1–42_. Cells were detached by treatment with 0.25% trypsin and collected by centrifugation for 5 min at 300 × *g*. The cells were then stained with an APC-labeled anti-CD11b antibody, PE-labeled anti-CD45 antibody, and PE-Cy7-labeled CD39 antibody (BioLegend). The geometric mean fluorescence intensity (MFI) of HF488-Aβ_1–42_ in the brain microglial cells (defined as CD11b^+^ CD45^+^ CD39^+^) [25] was measured using a Gallios flow cytometer with Kaluza software (Beckman Coulter, Inc., Brea, CA, USA). The internalized fluorescence of HF488-Aβ_1–42_ was evaluated as follows: (MFI of the sample without CyD treatment)—(MFI of the sample with CyD treatment). The relative phagocytic activity was calculated by dividing the internalized HF488-Aβ_1–42_ fluorescence of the sample by the average value of that activity in the NC group.

### Statistical analysis

Data are reported as mean ± SEM. All statistical analyses were performed using GraphPad Prism version 7.0 software (Graphpad Software Inc., La Jolla, CA, USA). Statistical differences between curves were analyzed using two-way ANOVA followed by Tukey’s multiple-comparisons test. Statistical differences among multiple groups were determined using one-way ANOVA followed by Tukey’s multiple-comparisons test. A probability (*p*) value of less than 0.05 was considered statistically significant.

## Results

### General observations

To determine whether oral administration of LPSp is effective in preventing the development of AD, we fed SAMP8 mice an HFD to induce experimental AD and drinking water spiked with purified LPSp for 18 weeks (Fig 1). Mice received LPSp at a dose of 0.3 or 1 mg/kg BW·day based on average BW and water intake during the experiment. As shown in Table 1, HFD-fed mice exhibited a significant increase in the final BW, daily weight gain, and food intake compared with LFD-fed (NC) mice (*p* < 0.05). The caloric intake in the HFD group was approximately 1.5 times as high as that in the NC group (*p* < 0.01). We found a significant increase in the weight of the liver and epididymal white adipose tissue (EWAT) in the HFD group (*p* < 0.05 vs. NC). When LPSp was orally administered to HFD-fed mice, no changes in final BW, daily weight gain, or food intake were observed, but significant reductions were seen in liver and EWAT weight compared with untreated HFD-fed mice (*p* < 0.05). There were no significant differences in initial BW among groups. During the experiment, no mouse died, and we saw no adverse reactions, such as rectal bleeding, diarrhea, or abnormal behavior.

**Table 1:** Body and organ weight changes over the experiment.

	Group			
	NC	HFD	HFD + LPSp 0.3 mg/kg	HFD + LPSp 1 mg/kg
Initial BW (g)	30.4 ± 1.6 ^a^	30.0 ± 0.9 ^a^	27.7 ± 1.0 ^a^	28.5 ± 1.5 ^a^
Final BW (g)	37.7 ± 1.5 ^a^	44.7 ± 0.8 ^b^	41.9 ± 1.5 ^b^	43.6 ± 1.7 ^b^
Daily weight gain (g/day)	0.06 ± 0.01 ^a^	0.12 ± 0.01 ^b^	0.11 ± 0.01 ^b^	0.12 ± 0.01 ^b^
Food intake				
(g/day)	3.6 ± 0.1 ^a^	4.1 ± 0.1 ^b^	4.1 ± 0.1 ^b^	4.0 ± 0.1 ^b^
(kJ/day)	57.1 ± 0.8 ^a^	87.9 ± 2.1 ^b^	88.9 ± 2.0 ^b^	86.3 ± 1.7 ^b^
Liver weight (g)	1.7 ± 0.1 ^a^	2.4 ± 0.1 ^b^	1.8 ± 0.1 ^a^	1.9 ± 0.1 ^a^
EWAT weight (g)	1.0 ± 0.1 ^a^	1.3 ± 0.1 ^b^	1.0 ± 0.1 ^a^	1.1 ± 0.1 ^a^

NC, negative control; HFD, high-fat diet; LPSp, lipopolysaccharide from *Pantoea agglomerans*; BW, body weight; EWAT, epidydimal white adipose tissue. Differences in letters (e.g., a, b) indicate statistically significant differences from each other (*p* < 0.05).

### Orally administered LPSp prevents HFD-induced impairment of glucose/lipid metabolism

**Fig 2:**
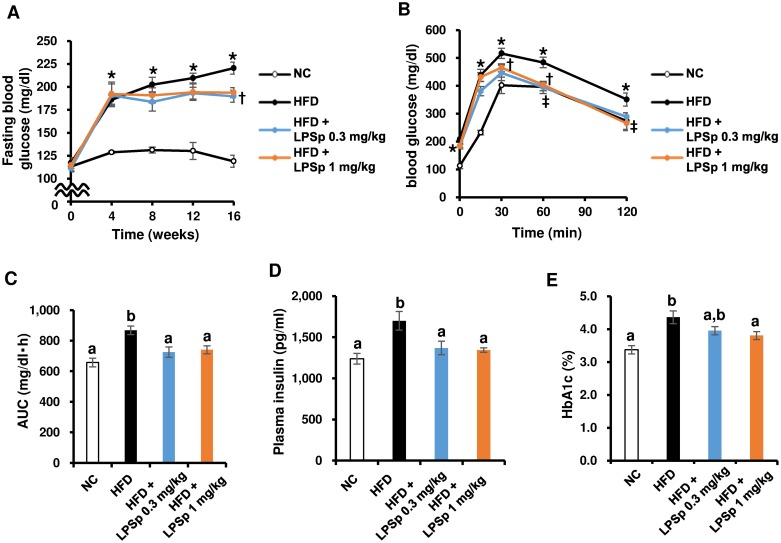
Effects of orally administered LPSp on fasting blood glucose, OGTT response, plasma insulin, and HbA1c. (A) The fasting glucose level in blood obtained from the tail vein is measured at every 4th week (weeks 0, 4, 8, 12, and 16). (B–C) In order to evaluate glucose tolerance, an oral glucose tolerance test (OGTT) was performed at week 17. The blood glucose level was measured at 0, 15, 30, 60, and 120 min after glucose loading (2 g _D_-glucose/kg BW) and AUC was calculated using the trapezoid rule. (D–E) The plasma insulin and HbA1c levels were measured after the experiment. Values are presented as the mean ± SEM, *n* = 4–7. * *p* < 0.05 for HFD vs. NC; † *p* < 0.05 for HFD + LPS 0.3 mg/kg vs. HFD; ‡ *p* < 0.05 for HFD + LPS 1mg/kg vs. HFD (two-way ANOVA followed by Tukey’s multiple-comparisons test). Unless indicated, no significance difference is observed between groups. Differences in letters between bars (*e*.*g*., a, b) indicate statistically significant differences between groups (*p* < 0.05, one-way ANOVA followed by Tukey’s multiple-comparisons test).

Chronic HFD feeding of mice induces an impairment of glucose and lipid metabolism, which triggers the development of obesity and its related metabolic disorders. Thus, we performed a fasting blood glucose measurement every four weeks (weeks 0, 4, 8, 12, and 16), and an oral glucose tolerance test (OGTT) at week 17. As expected, HFD-fed mice exhibited a marked elevation in the fasting blood glucose level from week 4 to 16 (*p* < 0.01 vs. NC, Fig 2A). There were no significant differences among groups in the fasting glucose level at week 0. As shown in Fig 2B and 2C, HFD-fed mice exhibited a pronounced increase in the blood-glucose response to a glucose load at 0, 15, 30, 60, and 120 min (*p* < 0.01 vs. NC) and in the area under the glucose curve (AUC; *p* < 0.01 vs. NC). In addition, the fasting plasma insulin (Fig 2D) and HbA1c (Fig 2E) levels were significantly higher in HFD-fed mice (all *p* < 0.01 vs. NC). Interestingly, when LPSp was orally administered to HFD-fed mice, the fasting blood glucose level at week 16 (Fig 2A) was significantly decreased with the lower dose of LPSp (0.3 mg/kg BW·day; *p* < 0.05 vs. HFD) and slightly decreased with the higher dose (1 mg/kg BW·day; *p* = 0.08). The OGTT analysis (Fig 2B) also showed that the lower dose of LPSp induced a significant reduction in the blood glucose level response at 30 and 60 min (*p* < 0.05 vs. HFD), whereas the higher dose of LPSp showed a significant reduction at 60 and 120 min (*p* < 0.05). Likewise, there was a significant reduction in the glucose AUC in both groups of LPSp-treated mice (both *p* < 0.05 vs. HFD) (Fig 2C). In addition, there was a significant decrease in the insulin level in both LPSp-treated groups (Fig 2D) and in HbA1c at the higher dose (Fig 2E) (all *p* < 0.05 vs. HFD).

**Fig 3:**
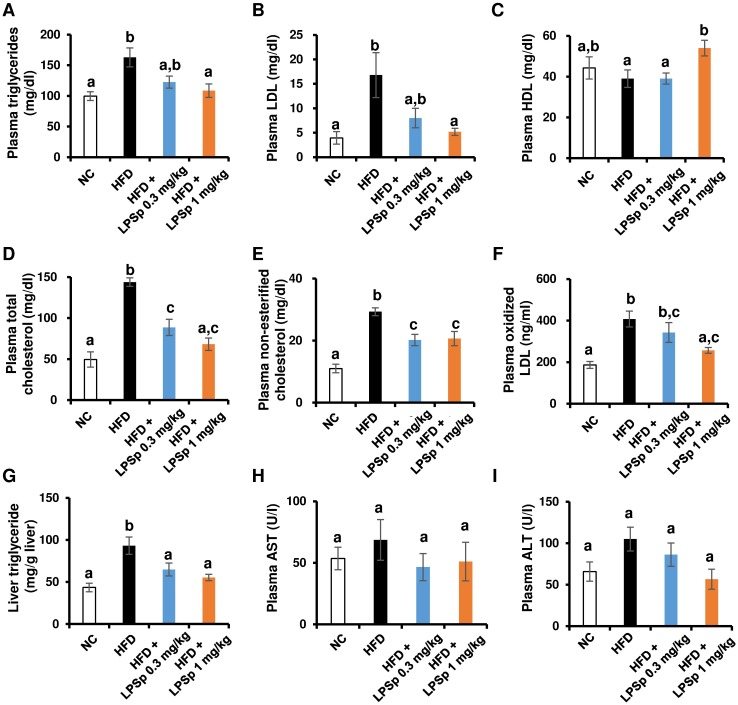
Effects of orally administered LPSp on plasma lipid profiles and hepatic triglyceride accumulation. (A–I) After the experimental period, plasma triglyceride, LDL, HDL, total cholesterol, non-esterified cholesterol, AST and ALT levels were measured using commercial kits. The hepatic triglyceride level was indicated as mg of triglyceride per g of liver weight. Values are presented as the mean ± SEM, *n* = 4–7. Differences in letters between bars indicate statistically significant differences between groups (*p* < 0.05, one-way ANOVA followed by Tukey’s multiple-comparisons test).

Next, to evaluate possible HFD-induced impairments of lipid metabolism in SAMP8 mice, we analyzed the biochemical parameters after the experiment. As shown in Fig 3A–3F, HFD-fed mice exhibited a significant increase in plasma triglycerides (*p* < 0.05 vs. NC), LDL (*p* < 0.05), total cholesterol (*p* < 0.01), non-esterified cholesterol (*p* < 0.01), and oxidized LDL (ox-LDL; *p* < 0.01), whereas no significant change was observed in plasma HDL level. The liver plays an important role in the maintenance of normal glucose/lipid metabolism, and HFD-induced impairment of liver function is characterized by the accumulation of hepatic lipid or elevation of plasma enzyme activities such as AST and ALT [26]. As shown in Fig 3G–3I, HFD feeding significantly induced hepatic triglyceride accumulation (*p* < 0.01 vs. NC) and tended to increase the activity of AST and ALT, but the latter effects did not reach significance in this experiment. In HFD-fed mice, the higher dose of LPSp was effective in lowering plasma triglycerides (*p* < 0.05), LDL (*p* < 0.05), total cholesterol (*p* < 0.01), and hepatic lipid accumulation (*p* < 0.01, all vs. HFD), to the extent of the levels seen in NC mice (Fig 3). Also, we saw a significant reduction in non-esterified cholesterol (*p* < 0.05) and a significant increase in HDL (*p* < 0.05, both vs. HFD). At the lower dose of LPSp, we found a significant reduction in plasma total cholesterol (*p* < 0.01 vs. HFD), non-esterified cholesterol (*p* < 0.05), and hepatic triglyceride (*p* < 0.05), and a slight reduction in plasma triglyceride (*p* = 0.08) and LDL (*p* = 0.10). Interestingly, the mice treated with the higher dose of LPSp exhibited a significant (*p* < 0.05) decrease in plasma ox-LDL compared with the HFD-fed mice (Fig 3F), suggesting that orally administered LPSp might prevent ox-LDL production and ox-LDL–mediated inflammatory responses *via* a reduction of oxidative stress in the peripheral blood. Taken together, these findings indicate that orally administered LPSp ameliorated the impairment of glucose/lipid metabolism induced by HFD intake in SAMP8 mice.

### Orally administered LPSp reduces HFD-induced inflammatory responses in the colon

**Fig 4:**
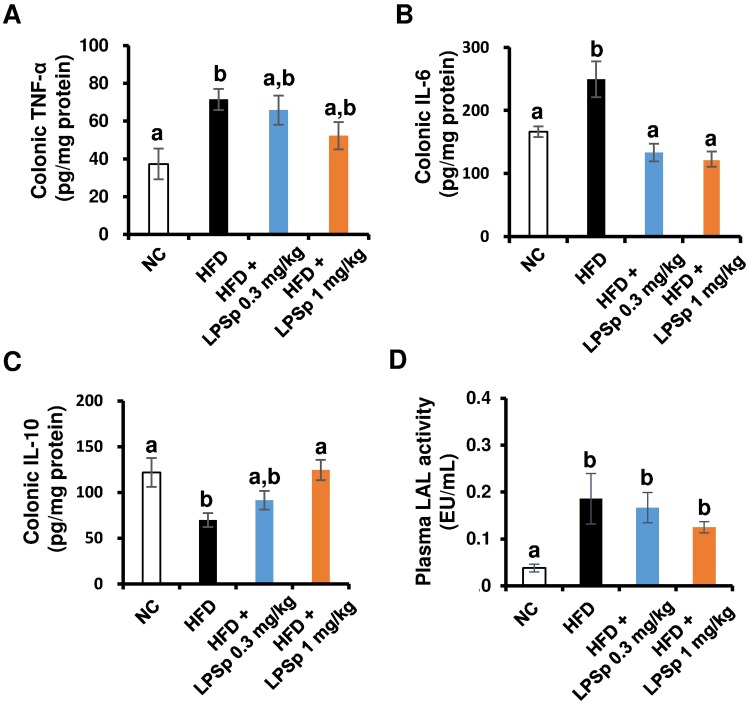
Effects of orally administered LPSp on colonic inflammatory mediators and total plasma LPS. (A–C) The concentration of TNF-α, IL-6 and IL-10 in the colon were determined using commercial ELISA kits. The concentration of cytokine is calculated as picograms of cytokine relative to protein content, *n* = 4–8. (D) The LAL assay was performed to evaluate the total abundance of LPS in the plasma, *n* = 3. Values are presented as the mean ± SEM. Differences in letters indicate statistically significant differences between groups (*p* < 0.05, one-way ANOVA followed by Tukey’s multiple-comparisons test).

In the early stages of the dysregulation of glucose/lipid metabolism caused by HFD feeding in rodents, intestinal inflammatory responses are triggered with an elevation of intestinal permeability, which increases the translocation of fatty acids and gut microbiome-derived LPS into the bloodstream, resulting in an aggravation of systemic inflammatory and oxidative stress [27]. Cytokine analysis of the colon tissue (Fig 4A–4C) showed that the HFD-fed mice had a significant increase in expression of the pro-inflammatory cytokines, TNF-α and IL-6, and a decrease in the anti-inflammatory cytokine IL-10 (all *p* < 0.05 vs. NC). The higher dose of LPSp was more effective in improving the HFD-induced IL-6 elevation and IL-10 reduction (both *p* < 0.01 vs. HFD), whereas no significant change was observed in TNF-α expression. In addition, the plasma LPS was measured using a LAL coagulation assay, which is the most widely used assay for detecting LPS. As shown in Fig 4D, the HFD-fed mice exhibited a significant increase in the plasma LPS (*p* < 0.05 vs. NC), suggesting that microbiome-derived LPS translocation into the bloodstream was enhanced by HFD treatment. The LPSp-treated mice showed no significant differences in the plasma LAL activity to the HFD-fed mice. These data suggest that orally administered LPSp might reduce HFD-induced intestinal inflammatory responses, but not affect the plasma level of LPS.

### Orally administered LPSp prevents HFD-induced inflammatory responses and oxidative-burst activities

**Fig 5:**
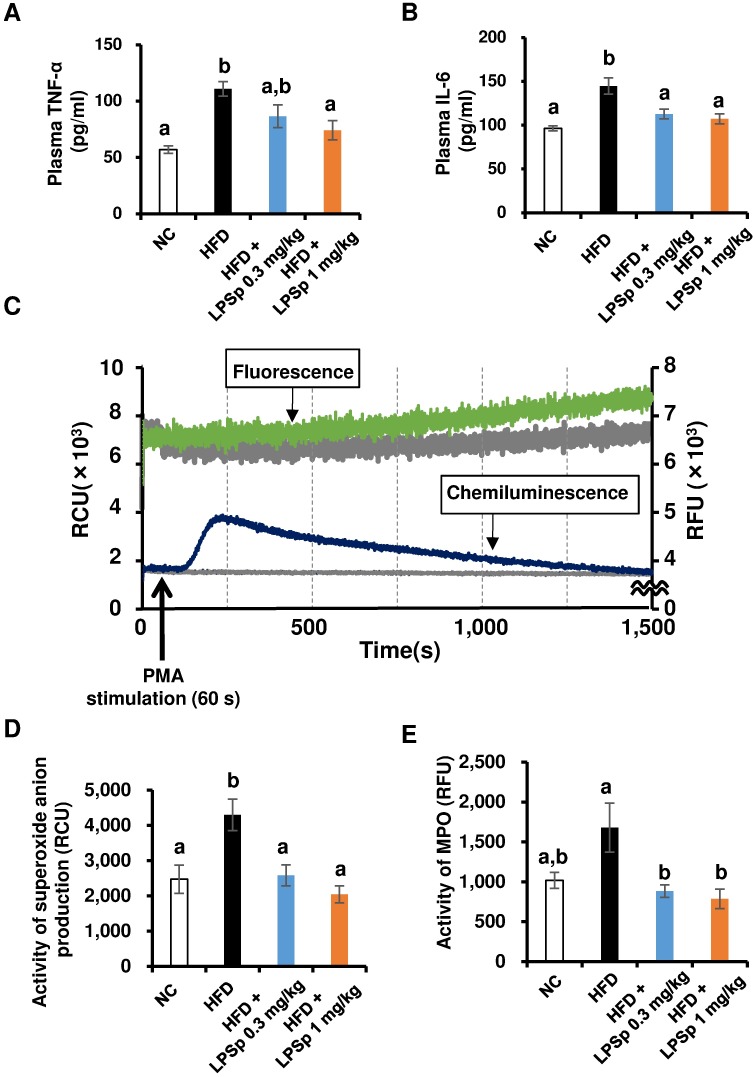
Effects of orally administered LPSp on two pro-inflammatory plasma cytokines and PMA-stimulated O2·- production and MPO activity in peripheral blood. (A–B) The plasma levels of TNF-α and IL-6 were determined using commercial ELISA kits, *n* = 4–7. (C) Representative time-change graphs of chemiluminescence (blue) and fluorescence (green) in the leukocyte fraction obtained from blood sample following stimulation by PMA. The negative controls are shown as gray lines. RCU, relative chemiluminescence units; RFU, relative fluorescence units. (D) O_2_^·-^ production and (E) MPO activity after PMA stimulation were both calculated by subtracting the basal intensity from the peak intensity. Values are presented as the mean ± SEM, *n* = 4–7. Differences in letters between bars indicate statistically significant differences between groups (*p* < 0.05, one-way ANOVA followed by Tukey’s multiple-comparisons test).

Next, we investigated whether orally administered LPSp is effective in regulating systemic inflammatory and oxidative responses. Cytokine analysis of the plasma (Fig 5A and 5B) found a remarkable increase in TNF-α and IL-6 in HFD-fed mice (both *p* < 0.01 vs. NC), whereas the higher dose of LPSp significantly reduced TNF-α and IL-6 expression (both *p* < 0.05 vs. HFD). The lower dose of LPSp produced a significant decrease in IL-6 (*p* < 0.05 vs. HFD). We examined oxidative-burst activity in the peripheral blood using a dual chemiluminescence/fluorescence spectrometer. The leukocyte fraction obtained from the peripheral blood was stimulated with PMA to induce O_2_^**·**-^ (superoxide anion) production and MPO reaction. Representative results from this device are shown in Fig 5C (O_2_^**·**-^: blue-colored line, MPO: green-colored line). The specificity of both chemiluminescence and fluorescence responses was confirmed by tests run in the presence of specific inhibitors (shown as gray-colored lines in Fig 5C) using SOD as a potent scavenger of O_2_^**·**-^ and 4-ABAH as a potent inhibitor of the MPO reaction. As shown in Fig 5D, HFD-fed mice exhibited a remarkable increase in PMA-stimulated O_2_^**·**-^ production (*p* < 0.05 vs. NC). In addition, the MPO activity in HFD-fed mice was *ca*. 1.6 times as high as that in NC mice (Fig 5E), but a significant difference was not observed here (*p* = 0.12). Orally administered LPSp was effective in lowering the HFD-induced elevation of O_2_^**·**-^ production and MPO activity (both *p* < 0.05). The proportion of neutrophils and monocytes in the blood was analyzed using a flow cytometer, and we found no significant differences among the groups in the proportion of neutrophils (defined as CD45^+^ CD11b^+^ Ly-6G^+^ cells) or monocytes (defined as CD45^+^ CD11b^+^ Ly-6G^-^ Ly-6C^+^ cells) (S1 Fig). Taken together, these results demonstrate that in HFD-fed mice, orally administered LPSp is effective in reducing plasma expression of pro-inflammatory cytokines and in decreasing the PMA-stimulated oxidative-burst activities.

### Orally administered LPSp ameliorates the HFD-induced accumulation of Aβ peptides in the brain and impairment of memory function

**Fig 6:**
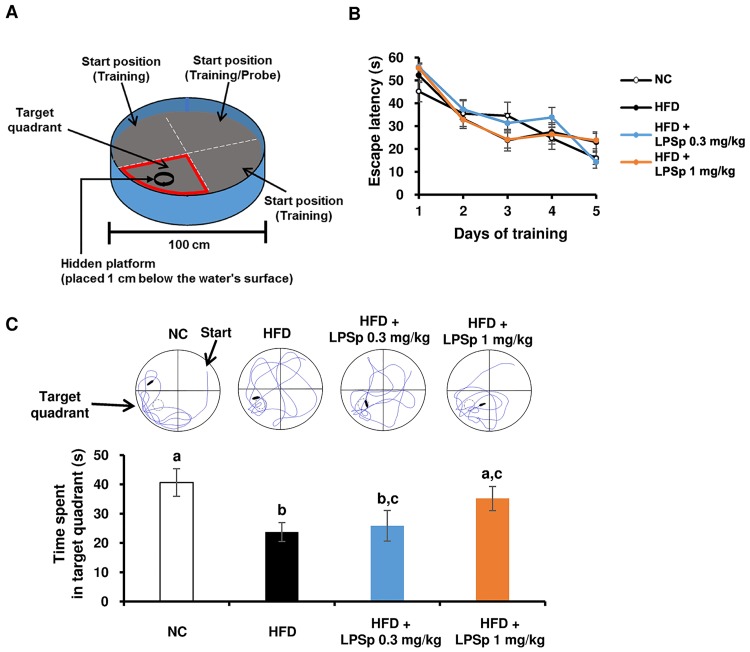
Effects of orally administered LPSp on cognitive performance as assessed by the Morris water maze. (A) Diagram of the water maze showing the position of the escape platform (black circle). The pool (100 cm in diameter, 40 cm in height) was filled with water, maintained at 23 ± 1°C, and rendered opaque with non-toxic black ink. A platform 10 cm in diameter was submerged 1 cm below the water surface in the middle of the target quadrant. The crossed, dashed lines indicate an imaginary division of the pool into four quadrants for purposes of analysis. Trial start positions are shown in each quadrant. (B) Training sessions continued over five days to assess learning ability. Each mouse was given 60 s to find the platform and was allowed to stay on it for 20 s. The amount of time elapsed before the mouse located the platform was defined as escape latency, and this latency was recorded as 60 s if the mouse failed to find the platform within 60 s. Each mouse was administered four trials per day. (C) The probe test was performed to assess memory ability. Each mouse was allowed to swim freely for 60 s in the pool without the platform. The time spent swimming in the target quadrant was recorded. The representative trajectory for each group is shown. Values are presented as the mean ± SEM, *n* = 4–6. Differences in letters indicate statistically significant differences between groups (*p* < 0.05, one-way ANOVA followed by Tukey’s multiple-comparisons test).

To examine whether orally administered LPSp improves cognitive functions in HFD-fed mice, we employed the Morris water maze (MWM) test, originally established to assess spatial learning and memory in rodents [28]. Fig 6A shows the diagram of MWM test in the present study. In the training sessions, mice learned the location of an escape platform for five successive days. As shown in Fig 6B, all animal groups were able to learn the position of the platform over the trial days, as indicated by a reduction in escape latency by approximately 50%, but a significant difference among groups was not observed. After the training session, the probe session was performed to assess memory performance. Each mouse was allowed to swim freely for 60 s in the pool without the platform, and the swimming trajectory and the time spent in the target quadrant were recorded. As shown in Fig 6C, HFD-fed mice exhibited a significant shortening of the time spent in the target quadrant compared with NC mice (*p* < 0.01), indicating that chronic intake of the HFD induced a decline in memory performance. Interestingly, the higher-dose LPSp treatment led to a significantly longer time spent in the target quadrant compared with the HFD-fed mice (*p* < 0.05). The representative swimming trajectories (Fig 6C) also indicated that the LPSp-treated mice exhibited a preference for swimming in the target quadrant. The results of the MWM test suggest that orally administered LPSp ameliorates HFD-induced memory deficits, but does not affect learning performance.

**Fig 7:**
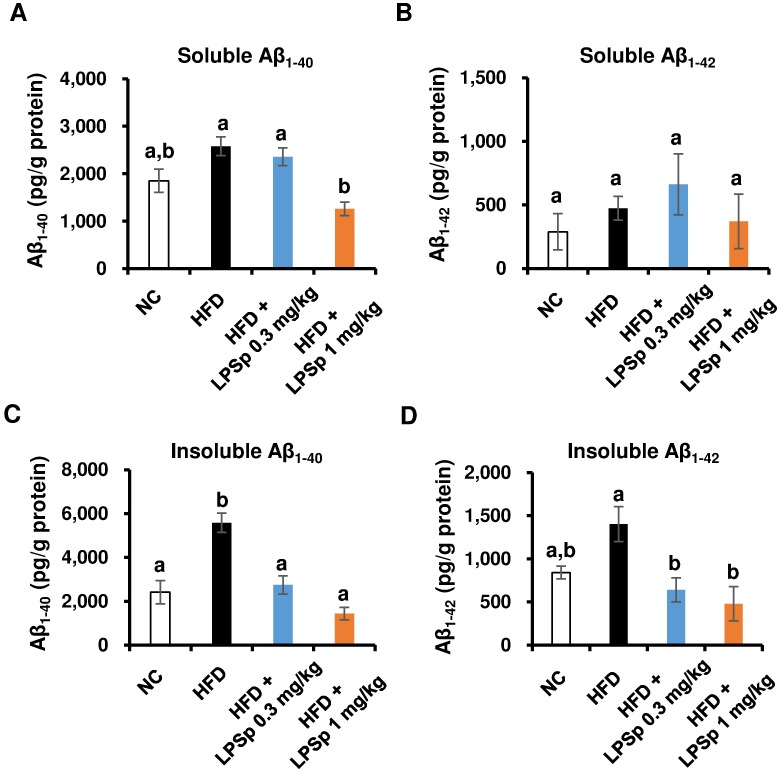
Effects of orally administered LPSp on the accumulation of Aβ_1–40_ and Aβ_1–42_ in the brain. The brain concentrations of two soluble Aβs (A, B) and insoluble Aβs (C,D) are shown. Values are presented as the mean ± SEM, *n* = 3–4. Differences in letters indicate statistically significant differences between groups (*p* < 0.05, one-way ANOVA followed by Tukey’s multiple-comparisons test).

We further investigated the effects of orally administered LPSp on the Aβ burden in the brains of HFD-fed mice. Both soluble and insoluble Aβ_1–40_ and Aβ_1–42_ levels in the brain were determined using ELISA kits. As shown in Fig 7A–7D, the HFD-fed mice exhibited a significant elevation in insoluble Aβ_1–40_ (*p* < 0.01) and a trend towards an increase in soluble Aβ_1–40_ (*p* = 0.10) and insoluble Aβ_1–42_ (*p* = 0.15) compared with the NC mice. The higher-dose LPSp treatment significantly reduced the accumulation of soluble Aβ_1–40_ (*p* < 0.01), insoluble Aβ_1–40_ (*p* < 0.01), and insoluble Aβ_1–42_ (*p* < 0.05) compared with HFD-fed mice. At the lower dose of LPSp, we found significant decreases in the levels of insoluble Aβ_1–40_ and Aβ_1–42_ (*p* < 0.01, *p* < 0.05 vs. HFD, respectively).

**Fig 8:**
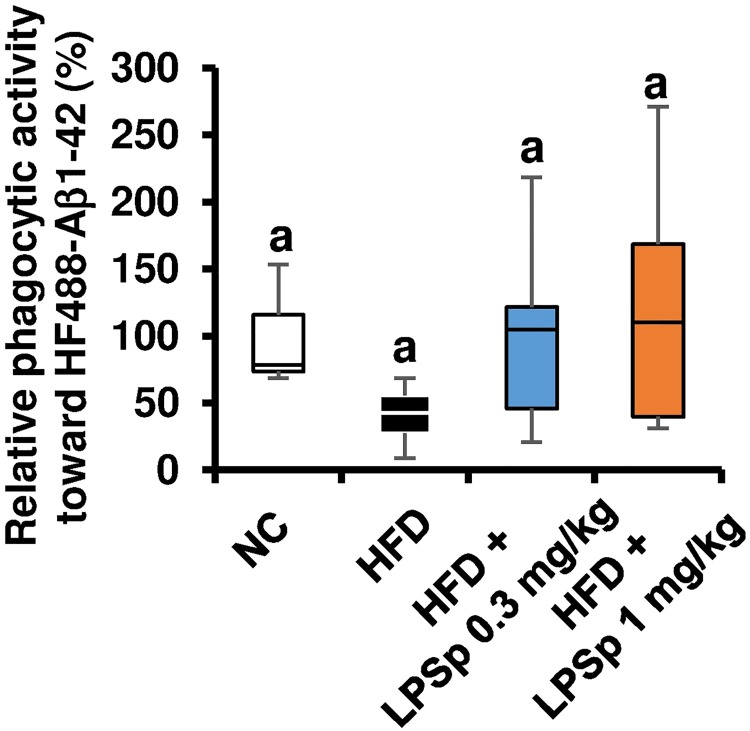
Effects of orally administered LPSp on phagocytic activity against Aβ_1–42_ in microglial cells isolated from the brain. The brains were collected after the experiment and the microglial cells isolated by the enzymatic digestion. The phagocytic activity against HF488- Aβ_1–42_ in the microglial cells was measured using a flow cytometer as described in the Methods. No significant difference is observed between groups (one-way ANOVA followed by Tukey’s multiple-comparisons test). The box-whisker plots describe the minimum (end of the bottom whisker), the first quartile (bottom border of the box), the median (line through the box), the third quartile (top border of the box), and the maximum (end of the top whisker) of the distribution, *n* = 3–6.

In addition, we attempted to investigate the underlying mechanisms of the observed preventative effects of oral LPSp on HFD-induced Aβ accumulation in the brain. We evaluated phagocytic activity against Aβ_1–42_ by isolated microglial cells. CD11b^+^ cells were isolated from the mouse brains after the experiment and then incubated with HiLyte^™^ Fluor 488-Aβ_1–42_ for 3 h. Using a flow cytometer, phagocytic activity toward Aβ_1–42_ was calculated from the fluorescence intensity of Aβ_1–42_ in CD11b^+^ CD45^+^ CD39^+^ microglial cells. As shown in Fig 8, HFD-fed mice exhibited a decrease in Aβ_1–42_ phagocytic activity by approximately 50% compared with the NC mice. The LPSp-treated mice showed a trend towards an increase in phagocytosis compared with the HFD-fed mice. However, we found no significant differences among groups, partly due to relatively high variation in the LPSp-treated mice. These findings demonstrate that orally administered LPSp prevents HFD-induced memory deficits and Aβ deposits in the brain, which seems to be associated with an improvement in glucose/lipid metabolism, an inhibition of inflammatory responses, and a reduction in oxidative-burst activity.

## Discussion

The present study showed that oral administration of LPSp for 18 weeks significantly prevented a memory deficit in HFD-fed SAMP8 mice. This was associated with an improvement of glucose tolerance and lipid profiles. Although *in vivo* studies aimed at prevention or treatment of AD usually use transgenic mouse models of AD, these transgenic mice have been designed mainly for expressing specific gene mutations present in early-onset familial AD, which accounts for only a small percentage (< 1%) of AD patients. The majority of AD cases are termed late-onset, sporadic AD, which is caused by metabolic or non-genetic environmental factors [29]. A growing body of evidence indicates that SAMP8 mice exhibit AD-like pathology and cognitive decline that result from aging and oxidative stress without genetic factors that act proximally on AD etiology [30]. Considering that orally administered LPSp has a beneficial effect on glucose/lipid metabolism [16], the present study used SAMP8 mice as a mouse model of AD to evaluate LPSp effects on HFD-induced AD progression. Consistent with recent studies using an HFD-fed SAMP8 [21, 31], the present data demonstrated that chronic HFD feeding induces a significant increase in BW, caloric intake, fasting insulin level, HbA1c, and the glucose AUC in the OGTT, which is associated with an impairment of memory performance in the MWM test. Interestingly, when LPSp was orally administered to the HFD-fed mice, improvements in the glucose response, insulin levels, and HbA1c levels were seen, and prevention of the memory deficit was observed at the dose of 1 mg/kg BW·day. Although there were no significant differences in the escape latency between groups when the age of mice was 7 months at the present training trial, the initial escape latency of HFD-fed mice (52.2 ± 3.1s) was slightly longer than that of NC mice (45.2 ± 4.6 s) (S1 Table). Other study using SAMP8 mice (5 months of age) indicated that the memory performance of HFD-fed mice was declined in the probe test, whereas there was no significant difference in the escape latency between groups at the training [31]. Deficit of learning performance in SAMP8, as indicated by significant longer escape latency in training, is usually observed when the age was over 8 months [32], implying that the present HFD-fed SAMP8 mice might represent an early stage of cognitive dysfunction. In addition, biochemical analysis indicated that LPSp prevented the HFD-induced changes in plasma lipid profiles and accumulation of hepatic triacylglycerol, which seems to be associated with a reduction in the weight of the liver and EWAT. Other studies using non-transgenic rodent models of AD have observed a preventive effect on the HFD-induced cognitive deficit *via* an improvement of glucose/lipid metabolism by orally administered food compounds [33, 34]. These data suggest that orally administered LPSp might prevent the HFD-induced memory decline, at least in part by improvement of glucose/lipid metabolism.

Next, we investigated the effects of LPSp on intestinal inflammation in HFD-fed mice. The present data indicated that the higher dose of LPSp significantly reduced the expression of IL-6 and increased that of IL-10 in the colon tissue. IL-10 has been shown to play an important role in preserving intestinal barrier function [35]. These data suggest that orally administered LPSp might reduce the HFD-induced inflammation and/or improve a barrier function in the colon. In addition, the present LAL test showed that the HFD-fed mice exhibited a significant increase in the plasma LPS. This is consistent with a previous report that HFD feeding in mice promotes translocation of microbiome-derived LPS to bloodstream *via* gut-barrier dysfunction and inflammation [36]. The LPSp-treated mice showed no significant differences in the plasma LAL activity to the HFD-fed mice. However, the LAL assay is not able to distinguish between administered LPSp and endogenous LPS because the reactivity of LAL reagents is dependent on the presence of the lipid A portion that is commonly contained in most LPS molecules [37]. Therefore, the present test seems to be limited to evaluating the total abundance of plasma LPS and be not able to estimate the ratio of translocation of LPSp and endogenous LPS to bloodstream. HFD-induced intestinal inflammation was shown to be associated with alterations of gut microbial composition, including a decrease in phylum *Bacteroidetes* and an increase in phylum *Firmicutes* in mice. These changes contribute to induce obesity and subsequent development of chronic diseases such as diabetes and AD [38]. We further investigated microbiota profiles in the stool samples (S2 Fig and S1 Dataset). The HFD-fed mice slightly decreased the abundance of *Bacteroidetes* (18.4 ± 1.7%) and increased *Firmicutes* (66.4 ± 1.9%) compared with the NC mice (*Bacteroidetes*, 21.2 ± 5.6%; *Firmicutes*, 64.3 ± 3.9%). LPSp treatment (1 mg/kg BW·day) slightly restored the decreased abundance of *Bacteroidetes* (25.0 ± 3.1%) in the HFD-fed mice, suggesting LPSp treatment might regulate the microbiota profiles *via* an improvement of glucose/lipid metabolism and intestinal inflammation in HFD-fed mice. The genus *Bacteroides*, one predominant gram-negative enterobacterium, possesses LPS comprised of penta-acylated lipid A moiety. The *in vitro* potency of macrophage activation by endogenous *Bacteroides*-derived LPS is ~1,000-fold lower than *E*. *coli* or *Salmonella minnesota* LPS that contains hexa- or hepta-acylated lipid A [39]. The LPSp used here also possesses hexa- or hepta-acylated lipid A [40], and LPSp treatment activated gene expression of cytokines in a macrophage cell line to the same degree as *E*. *coli* LPS [41]. Oral administration of *E*.*coli* LPS was found to regulate a mucosal immunity *via* intestinal TLR4 signaling to enhance immune defense in the intestine without activating serum TNF-α expression in mice [42], suggesting that a beneficial immunomodulatory effect through gastrointestinal tract might be induced by orally treated LPS, not by microbiome-derived endogenous LPS. van Puijvelde et al. [43] demonstrated that an orally administrated HSP (acts as TLR 4 agonist) can activate T regulatory (Treg) cells in intestine and blood, resulting in a prevention of atherosclerosis in mice, but a relationship between Treg function and microbiota profiles was not clarified. Depletion of Treg cells accelerates cognitive dysfunction and decreases microglial activity in a mouse model of AD [44], suggesting Treg cells plays a critical role of AD pathology. Since chronic HFD treatment also contributes to alter Treg functions that is associated with impaired glucose/lipid metabolisms in mice [45], further study are required to clarify the effect of orally treated LPSp on intestinal Treg functions and its relationship with metabolic functions or microglial activity using a Treg-depleted mouse model of AD without HFD.

We further investigated the effects of LPSp on oxidative responses in HFD-fed mice. A recent meta-analysis of patients with AD or mild cognitive impairment indicates that abnormal oxidative stress in the blood is involved in the early stages of neurodegeneration, and accelerates AD progression [46]. In the oxidative-burst series of reactions, NADPH oxidase catalyzes the production of O_2_^**·**-^, which is then converted to hydrogen peroxide (H_2_O_2_) by the enzyme superoxide dismutase. The enzyme MPO then converts the H_2_O_2_ to hypochlorous acid, an even stronger oxidant [47]. In the present study, the oxidative burst activity in peripheral blood sample was assessed by quantifying two key activities: O_2_^**·**-^ production and MPO responses. The leukocyte fraction obtained from peripheral blood was stimulated with PMA to trigger O_2_^**·**-^ production and MPO responses. After stimulation, the chemiluminescence response indicating O_2_^**·**-^ production was observed first, and then the response gradually decreased. The fluorescence response, which indicated the MPO reaction, gradually increased over the measurement period (Fig 5C). The present assay seems to be preferred for monitoring a series of PMA-induced oxidative bursts in the same sample. The present data indicated that oral administration of LPSp produces a significant reduction in the HFD-induced increase in O_2_^**·**-^ production and MPO activity, without affecting the numbers of neutrophils and monocytes in the peripheral blood. This suggests that orally administered LPSp might inhibit a hyper-responsiveness to PMA on the peripheral leukocytes. Since exacerbation of oxidative bursts is associated with ox-LDL generation and ox-LDL-related inflammation [47], the lowering effect on plasma ox-LDL levels by LPSp might be mediated through a reduction in HFD-induced activation of oxidative bursts.

To further investigate the possible mechanisms of the preventative effect of LPSp on memory decline, accumulation of Aβ peptides in the brain was evaluated by ELISA. The present data indicate that HFD feeding results in a greater burden of insoluble Aβ peptides (Aβ_1–40_ and Aβ_1–42_), but not soluble ones, which is in agreement with some other studies using an HFD-fed mouse model of AD [48, 49]. Although the mechanisms of amyloidogenesis by Aβ peptides remain unclear, a growing body of evidence indicates that i) cleavage of APP produces the major Aβ peptides, including Aβ_1–40_ and Aβ_1–42_, ii) these Aβ peptides undergo self-polymerization, followed by formation of Aβ oligomers (soluble Aβ), and iii) Aβ oligomers aggregate to form Aβ fibrils or plaques (insoluble Aβ), considered a hallmark of AD [50]. Here, orally administered LPSp produced a significant reduction in soluble Aβ_1–40_, insoluble Aβ_1–40_, and insoluble Aβ_1–42_ in the HFD-fed mice, suggesting that LPSp could promote clearance of Aβ peptides. Therefore, we used microglial cells isolated from the brains after treatment for phagocytic activity against Aβ_1–42_. Although no significant intergroup differences were observed, some of our results suggest that HFD might lead to an impairment of the phagocytic activity, and the results with LPSp treatment seemed to show a trend towards recovery of this activity. The herbal medicine Juzen-taiho-to, which contains LPS as the major active compound [51], was shown to reduce brain Aβ burden by oral treatment in a mouse model of AD, and to enhance *in vitro* Aβ_1–42_ phagocytosis [52]. Some *in vivo* studies reported that the ratio of absorption into blood after oral administration of radiolabeled *E*.*coli* LPS was estimated to be *ca*. 0.3% with the aid of chylomicron [53] and *E*.*coli* LPS can reach into the brain at *ca*. 0.02% at 24 h after i.p. treatment [54]. Our previous *in vitro* studies showed that Aβ_1–42_ phagocytosis in mouse microglia was enhanced by LPSp treatment as low as at 10 ng/ml [19, 20]. These findings support the hypothesis that oral LPSp treatment could be effective in promoting *in vivo* Aβ phagocytosis. Since few studies have demonstrated an *in vivo* enhancement of Aβ phagocytosis after oral treatment of anti-AD agents, further studies are needed to elucidate the pharmacokinetics of orally administered LPSp and *in vivo* mechanisms underlying brain Aβ clearance by LPSp treatment.

To the best of our knowledge, this preliminary study provides new evidence that oral administration of LPSp significantly inhibits the HFD-induced decline in memory performance and accumulation of brain Aβ peptides in SAMP8 mice. These effects were associated with improvement of glucose/lipid metabolism and a reduction of inflammatory/oxidative responses; however we could not confirm a significant enhancement of Aβ phagocytosis in the isolated microglia after treatment. Based on our findings, we propose that LPSp holds promise as a potential preventative compound for AD or AD-related diseases induced by impairment of metabolic functions.

## Supporting information

S1 Fig The population of neutrophils and monocytes in the peripheral blood sample. The population of (A) CD45^+^CD11b^+^Ly-6G^+^ neutrophils and (B) CD45^+^CD11b^+^Ly-6G^-^Ly-6C^+^ monocytes are indicated as the percentage of CD45^+^CD11b^+^ cells as described in the supporting information. Values are presented as the mean ± SEM, *n* = 4–7. No significant difference is observed between groups (one-way ANOVA followed by Tukey’s multiple-comparisons test).
(PDF)

S2 Fig Gut microbiota composition. The microbiota profiles in the stool were analyzed as described in the supporting information. (A) Variation in bacterial community compositions in each stool sample at the phylum levels. Right 4 lanes indicate the average value of each group. (B) The relative abundance (%) of phylum *Bacteroidetes* and *Firmicutes*. Values are presented as the mean ± SEM, *n* = 4. No significant difference is observed between groups (one-way ANOVA followed by Tukey’s multiple-comparisons test).
(PDF)

S1 Table The escape latency in the training session of MWM test. NC, negative control mice; HFD, high-fat diet-fed mice; LPSp, lipopolysaccharide from *Pantoea agglomerans*; Values are presented as the mean ± SEM (s).
(DOCX)

S1 File Supplementary methods. (DOCX)

S1 Dataset Taxonomy summary dataset of microbiota analysis. (ZIP)
