# Supplementary figures and images for: Oral administration of Pantoea agglomerans-derived lipopolysaccharide prevents metabolic dysfunction and Alzheimer’s disease-related memory loss in senescence-accelerated prone 8 (SAMP8) mice fed a high-fat diet
Source: PLoS One. 2018 Jun 1;13(6):e0198493. doi: 10.1371/journal.pone.0198493 (PMC5983504; doi:10.1371/journal.pone.0198493)

**A**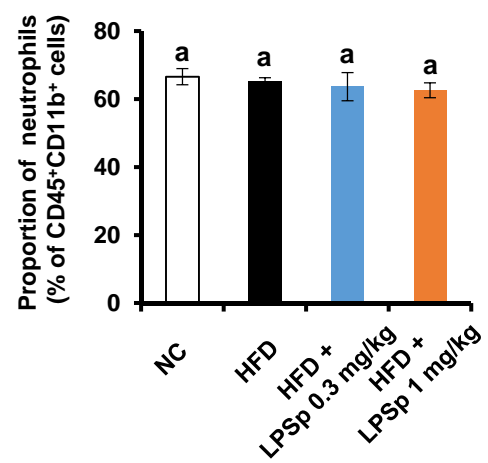**B**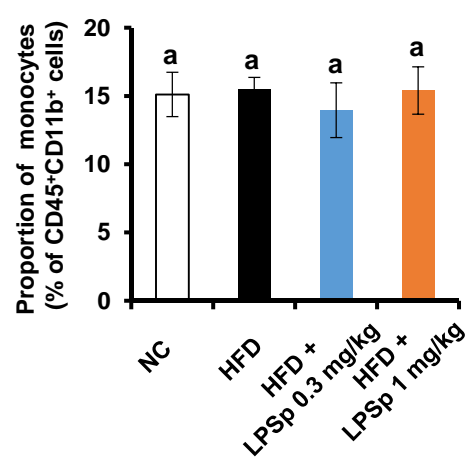

Supplement: S1 Fig — The population of (A) CD45+CD11b+Ly-6G+ neutrophils and (B) CD45+CD11b+Ly-6G-Ly-6C+ monocytes are indicated as the percentage of CD45+CD11b+ cells as described in the supporting information. Values are presented as the mean ± SEM, n = 4–7. No significant difference is observed between groups (one-way ANOVA followed by Tukey’s multiple-comparisons test). (PDF) [file pone.0198493.s001.pdf]

**A**

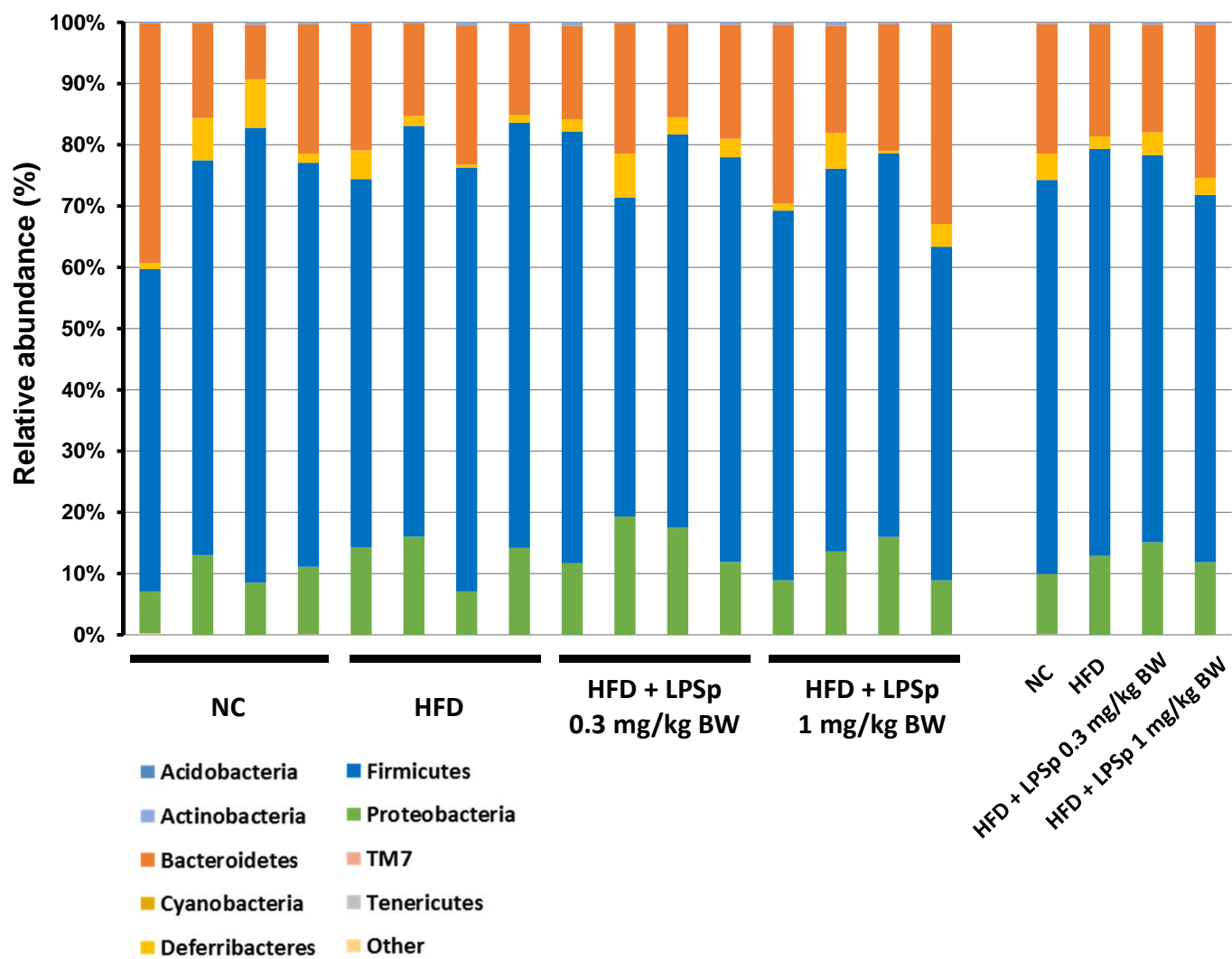

**B**

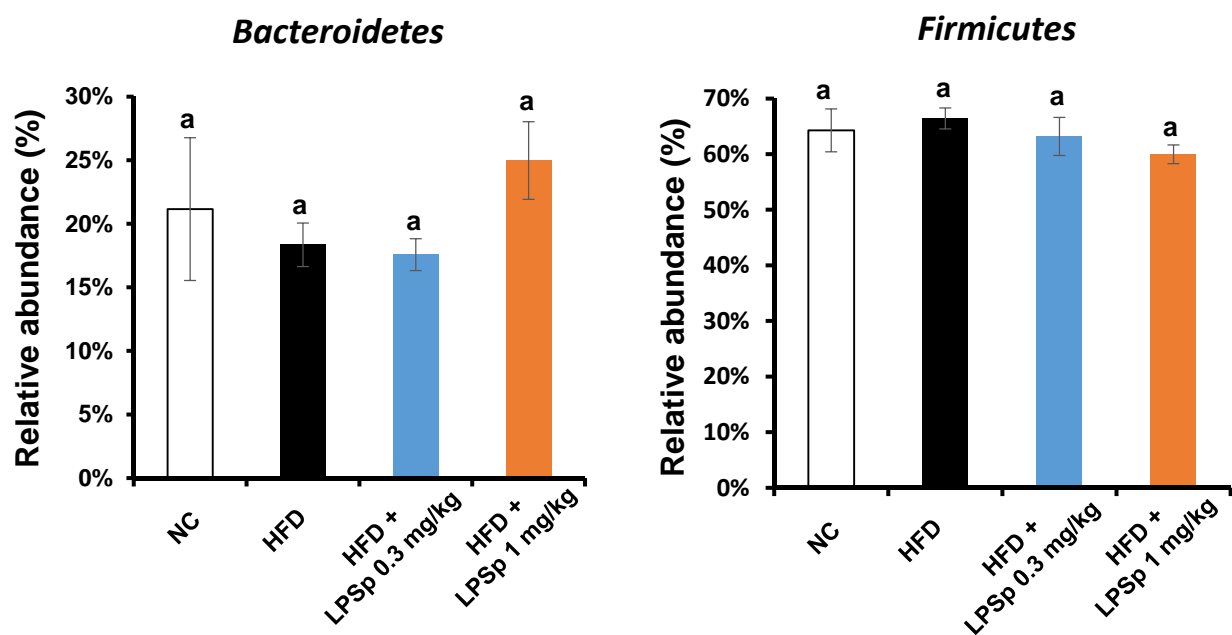

Supplement: S2 Fig — The microbiota profiles in the stool were analyzed as described in the supporting information. (A) Variation in bacterial community compositions in each stool sample at the phylum levels. Right 4 lanes indicate the average value of each group. (B) The relative abundance (%) of phylum Bacteroidetes and Firmicutes. Values are presented as the mean ± SEM, n = 4. No significant difference is observed between groups (one-way ANOVA followed by Tukey’s multiple-comparisons test). (PDF) [file pone.0198493.s002.pdf]
