# Supplementary material for: Oral administration of Pantoea agglomerans-derived lipopolysaccharide prevents metabolic dysfunction and Alzheimer’s disease-related memory loss in senescence-accelerated prone 8 (SAMP8) mice fed a high-fat diet
Source: PLoS One. 2018 Jun 1;13(6):e0198493. doi: 10.1371/journal.pone.0198493 (PMC5983504; doi:10.1371/journal.pone.0198493)
Supplement: S1 Table — NC, negative control mice; HFD, high-fat diet-fed mice; LPSp, lipopolysaccharide from Pantoea agglomerans; Values are presented as the mean ± SEM (s). (DOCX) [file pone.0198493.s003.docx]

| Group | Day 1 | | | Day 2 | | | Day 3 | | | Day 4 | | | Day 5 | | |
| --- | --- | --- | --- | --- | --- | --- | --- | --- | --- | --- | --- | --- | --- | --- | --- |
| NC | 45.2 | ± | 4.6 | 35.5 | ± | 5.6 | 34.5 | ± | 6.0 | 24.7 | ± | 4.9 | 15.9 | ± | 2.7 |
| HFD | 52.2 | ± | 3.1 | 33.2 | ± | 4.1 | 23.8 | ± | 4.7 | 27.4 | ± | 4.0 | 23.0 | ± | 3.8 |
| HFD+ LPSp  0.3 mg/kg | 55.9 | ± | 1.7 | 37.3 | ± | 4.3 | 31.3 | ± | 4.3 | 33.8 | ± | 4.3 | 14.3 | ± | 2.8 |
| HFD+ LPSp  1 mg/kg | 55.4 | ± | 1.7 | 32.8 | ± | 3.8 | 24.1 | ± | 3.7 | 26.4 | ± | 4.3 | 23.8 | ± | 3.7 |
