# Supplementary material for: Oral administration of Pantoea agglomerans-derived lipopolysaccharide prevents metabolic dysfunction and Alzheimer’s disease-related memory loss in senescence-accelerated prone 8 (SAMP8) mice fed a high-fat diet
Source: PLoS One. 2018 Jun 1;13(6):e0198493. doi: 10.1371/journal.pone.0198493 (PMC5983504; doi:10.1371/journal.pone.0198493)
Supplement: S1 File — (DOCX) [file pone.0198493.s004.docx]

**Supplementary Methods**

**Flow-cytometric analysis of neutrophils and monocytes in blood.** Thirty-microliter blood aliquots were incubated for 15 min on ice with each of the following antibodies (BioLegend, San Diego, CA, USA): PE-labelled anti-mouse CD45 (clone 30-F11), PE/Cy7 anti-mouse/human CD11b (clone M1/70), APC anti-mouse Ly-6G (clone 1A8), and APC/Fire750 anti-mouse Ly-6C (clone HK1.4). The stained blood samples were lysed in a red-blood-cell lysis buffer (Tonbo Biosciences, San Diego, CA, USA) and analyzed using a Gallios flow cytometer with Kaluza software (Beckman Coulter, Inc., Brea, CA, USA). The gated CD45^+^ CD11b^+^ cells were defined as total white blood cells, CD45^+^ CD11b^+^ Ly-6G^+^ as neutrophils, and CD45^+^ CD11b^+^ Ly-6G^-^ Ly-6C^+^ as monocytes [1]. The numbers of neutrophils and monocytes are reported as percentage of total CD45^+^ CD11b^+^ leukocytes.

**Microbiota analysis.** Stool sample was collected after the treatment and stored at −80°C until use. The extraction, amplification, sequencing and analysis of DNA were performed by Bioengineering Lab (Kanagawa, Japan). Briefly, microbial DNA was extracted from mouse stools using the MPure bacterial DNA extraction kit (MP Biomedicals, Santa Ana, CA, USA). The 16S rRNA V3-V4 region was amplified using 16S amplicon PCR primers (341f and 805r). Sequencing of the purified 16S rRNA amplicons was carried out on an Illumina Miseq platform (Illumina, Inc., San Diego, CA, USA). Post processing sequencing data were analysed with QIIME software [2]. Quality filtering was performed using the default parameters in QIIME. Sequences were grouped into operational taxonomic units at a 97% sequence similarity threshold.

**SI References**

1. Takehara M, Seike S, Takagishi T, Kobayashi K, Nagahama M. Peptidoglycan accelerates granulopoiesis through a TLR2- and MyD88-dependent pathway. Biochem Biophys Res Commun. 2017;487:419-425.

2. Caporaso JG, Kuczynski J, Stombaugh J, Bittinger K, Bushman FD, Costello EK et al. QIIME allows analysis of high-throughput community sequencing data. Nat Methods. 2010;7:335-336.
